# Ezrin Mediates Neuritogenesis via Down-Regulation of RhoA Activity in Cultured Cortical Neurons

**DOI:** 10.1371/journal.pone.0105435

**Published:** 2014-08-21

**Authors:** Yosuke Matsumoto, Masatoshi Inden, Atsushi Tamura, Ryo Hatano, Sachiko Tsukita, Shinji Asano

**Affiliations:** 1 Department of Molecular Physiology, College of Pharmaceutical Sciences, Ritsumeikan University, Kusatsu, Shiga, Japan; 2 Laboratory of Medical Therapeutics and Molecular Therapeutics, Gifu Pharmaceutical University, Gifu, Japan; 3 Laboratory of Biological Science, Graduate School of Frontier Biosciences and Graduate School of Medicine, Osaka University, Suita, Osaka, Japan; Osaka University Graduate School of Medicine, Japan

## Abstract

Neuronal morphogenesis is implicated in neuronal function and development with rearrangement of cytoskeletal organization. Ezrin, a member of Ezrin/Radixin/Moesin (ERM) proteins links between membrane proteins and actin cytoskeleton, and contributes to maintenance of cellular function and morphology. In cultured hippocampal neurons, suppression of both radixin and moesin showed deficits in growth cone morphology and neurite extensions. Down-regulation of ezrin using siRNA caused impairment of netrin-1-induced axon outgrowth in cultured cortical neurons. However, roles of ezrin in the neuronal morphogenesis of the cultured neurons have been poorly understood. In this report, we performed detailed studies on the roles of ezrin in the cultured cortical neurons prepared from the ezrin knockdown (*Vil2^kd/kd^*) mice embryo that showed a very small amount of ezrin expression compared with the wild-type (*Vil2^+/+^*) neurons. Ezrin was mainly expressed in cell body in the cultured cortical neurons. We demonstrated that the cultured cortical neurons prepared from the *Vil2^kd/kd^* mice embryo exhibited impairment of neuritogenesis. Moreover, we observed increased RhoA activity and phosphorylation of myosin light chain 2 (MLC2), as a downstream effector of RhoA in the *Vil2^kd/kd^* neurons. In addition, inhibition of Rho kinase and myosin II rescued the impairment of neuritogenesis in the *Vil2^kd/kd^* neurons. These data altogether suggest a novel role of ezrin in the neuritogenesis of the cultured cortical neurons through down-regulation of RhoA activity.

## Introduction

Establishment of neural circuits in the central nerve system requires generation and development of multiple dendrites and single axon. Cultured neurons showing a sequence of morphological changes have been well studied for neuronal morphogenesis [1], [2]. In particular, neuritogenesis that is the first step in neuronal morphogenesis is driven by exocytic and cytoskeletal machinery [3]. Several neurites extended from a symmetrical cell body become an axon or dendrites, and subsequently, neurons establish synaptic connections and networks. Small GTPases, RhoA, Rac1 and Cdc42 modulate the neuronal morphogenesis through regulating cytoskeletal dynamics in different pathways [4]. Rac1 and Cdc42 promote neurite outgrowth through phosphorylation of p21-activated kinase (PAK) family of serine/threonine kinases [5]. In contrast, RhoA and its downstream effector Rho kinase mediate neurite retractions [6]. Myosin II activity is determined by phosphorylation of myosin light chains (MLCs) and mediated by RhoA/Rho kinase pathway. Activated myosin II generates formation of cortical actin filaments and leads to inhibition of neuritogenesis [7].

Ezrin, radixin and moesin (ERM) proteins are membrane-cytoskeleton linkers and regulate Rho activity through interaction with Rho guanine nucleotide dissociation inhibitor or Rho GTPase-activating protein [8]-[10]. In the cultured hippocampal neurons, expression of the ERM proteins was first detected by mouse monoclonal 13H9 antibody that recognized all members of the ERM proteins [11]. Among the ERM proteins, radixin and moesin were enriched in growth cone structure and associated with neurite extensions in the cultured hippocampal neurons [12]. Ezrin was associated with axon outgrowth induced by netrin-1 stimulation [13], however, expression of ezrin was mainly detected in cell body [12]. Therefore, the role of ezrin in the neuronal morphogenesis has remained unclear.

In the present study, to examine the functions of ezrin in the neuronal morphogenesis, we newly used cultured cortical neurons prepared from ezrin knockdown (*Vil2^kd/kd^*) mice in which ezrin expression levels were decreased to less than 5% compared with the wild-type (*Vil2^+/+^*) mice [14]. The *Vil2^kd/kd^* mice showed achlorhydria due to impairment of membrane fusion between intracellular gastric vesicles and apical membrane in gastric parietal cells. In this report, the cultured cortical neurons prepared from the *Vil2^kd/kd^* mouse embryo showed reduction in number of neurites compared with the *Vil2^+/+^* neurons whereas length of neurites and axon was not changed. We studied whether RhoA, Rac1 and Cdc42 activities were modulated in the cultured cortical neurons prepared from the *Vil2^kd/kd^* mouse embryo. Treatment of Rho kinase inhibitor Y-27632 or myosin II inhibitor blebbistatin was reported to promote initiation of axon outgrowth and neuritogenesis, respectively [15]–[17]. We studied effects of these inhibitors on the neuritogenesis in the *Vil2^kd/kd^* neurons. Our data suggest that ezrin is a key player of the neuritogenesis in the cultured cortical neurons through down-regulation of the RhoA activity.

## Materials and Methods

### Mice

*Vil2^kd/kd^* mice were prepared as described previously [14]. All works with animals were performed with approval from the Animal Ethics Committee of Ritsumeikan University.

### Neuronal culture

Primary cortical neurons were prepared from littermate *Vil2^+/+^* and *Vil2^kd/kd^* mouse embryos (E15.5) as described previously [18]. Briefly, cortices were dissected and incubated with 0.25% (w/v) trypsin/EDTA for 20 min at 37°C. Cells were seeded onto culture dishes coated with poly-D-lysine and grown in Neurobasal Medium (Invitrogen) containing B27 supplement (Invitrogen), GlutaMAX supplement (Invitrogen), 0.3% glucose and 37.5 mM NaCl. More than 95% of cell populations were neurons in our culture condition. For drug treatments, cells were treated with 40 µM Y-27632 (Wako) and 50 µM blebbistatin (Wako) for indicated period after plating.

### Immunoblotting

3×10^6^ cells were seeded onto 60 mm culture dishes coated with poly-D-lysine and then lysed with RIPA (25 mM Tris-HCl pH 7.6, 150 mM NaCl, 1% Nonidet P-40, 1% sodium deoxycholate, 0.1% sodium dodecyl sulfate) buffer with protease inhibitors (Cell BioLabs) and phosphatase inhibitors (Nacalai Tesque). Cell lysate protein extracts were separated by SDS-PAGE and transferred to a polyvinylidene difluoride membrane. The membranes were blocked with 5% skim milk in TBST (10 mM Tris-HCl, pH 8.5, 150 mM NaCl and 0.1% Tween 20) solution, followed by incubation with the following primary antibodies overnight at 4°C. A rabbit anti-ezrin antibody (#3145, 1∶1000, Cell Signaling Technology), a rabbit anti-ERM antibody (#3142, pan-ERM, 1∶1000, Cell Signaling Technology), a rabbit anti-phospho ERM antibody (#3141, 1∶1000, Cell Signaling Technology), a rabbit anti-GAPDH antibody (1∶10000, Sigma), a rabbit anti-myosin light chain 2 (MLC2) antibody (#3672, 1∶100, Cell Signaling Technology), a rabbit anti-phospho MLC2 (Ser19) antibody (#3671, 1∶100, Cell Signaling Technology), a mouse anti-RhoA antibody (1∶500, Cytoskeleton), a mouse anti-Rac1 antibody (1∶500, Cell BioLabs), a mouse anti-Cdc42 antibody (1∶500, Cytoskeleton). After incubation with a horseradish peroxidase-conjugated goat anti-mouse or anti-rabbit IgG (Millipore) at room temperature for 1 h, image bands were detected by using Immobilon Western Chemiluminescent HRP Substrate (Millipore) and quantified using ImageJ software.

### Immunofluorescence

1×10^4^ cells were fixed with 4% paraformaldehyde and 4% sucrose in PBS for 10 min at 4°C. Fixed cells were permeabilized with 0.1% Triton X-100 in PBS for 10 min at room temperature. Cells were treated with 1% BSA in PBS for 30 min at room temperature, and incubated with primary antibodies overnight at 4°C, followed by the treatment with secondary antibodies for 45 min at room temperature. The primary antibodies used in this study were a mouse anti-α-tubulin antibody (1∶1000, DM1A, Abcam), a rabbit anti-ezrin antibody (#3145, 1∶100, Cell Signaling Technology), a rabbit anti-neuronal class III β-tubulin antibody (1∶1000, TUJ1, Covance). The secondary antibodies used in this study were a fluorescein isothiocyanate (FITC)-conjugated anti-rabbit IgG (Jackson ImmunoResearch) and an Alexa Fluor 633-conjugated anti-mouse IgG (Invitrogen). For filament actin staining, rhodamine phalloidin (Invitrogen) was added to secondary antibody. Fluorescence images were acquired with a confocal laser scanning microscope (FV-1000D, FV-10i, Olympus).

#### Rho activation assay

Cells were homogenized at 4°C in cell lysis buffer (25 mM HEPES, pH 7.5, 150 mM NaCl, 1% Nonidet P-40, 10 mM MgCl_2_, 2% glycerol) and centrifuged at 14,000×g for 10 min. Cell extracts were incubated with GST-rhotekin-RBD (Cytoskeleton) or GST-PAK-PBD (Cell BioLabs) fusion protein that had been conjugated with glutathione beads at 4°C for 1 h, and washed three times with the cell lysis buffer. GST-rhotekin-RBD-bound RhoA, and GST-PAK-PBD-bound Rac1 and Cdc42 were analyzed by SDS-PAGE, and subsequently immunoblotted with RhoA, Rac1 and Cdc42-specific antibodies, respectively.

### Morphological analysis

To categorize each stage of cells, cells were defined by the length of the longest neurite: stage 1 (nonpolar), <10 µm; stage 2 (multipolar), <40 µm; stage 3 (axon-forming), >40 µm as reported previously [19]. Axon was defined by the following criteria: a process more than twice longer than other processes [20]. The length and number of neurites and length of axon in each neuron were measured by manual tracing using ImageJ software with NeuronJ plugin. Neurons were identified by immunofluorescence with an anti-neuronal class III β-tubulin antibody.

### Statistical analysis

Results are expressed as mean ± SE. The significance of differences was determined by Student's t test.

## Results

### Ezrin knockdown showed impairment of neuritogenesis

**Figure 1:**
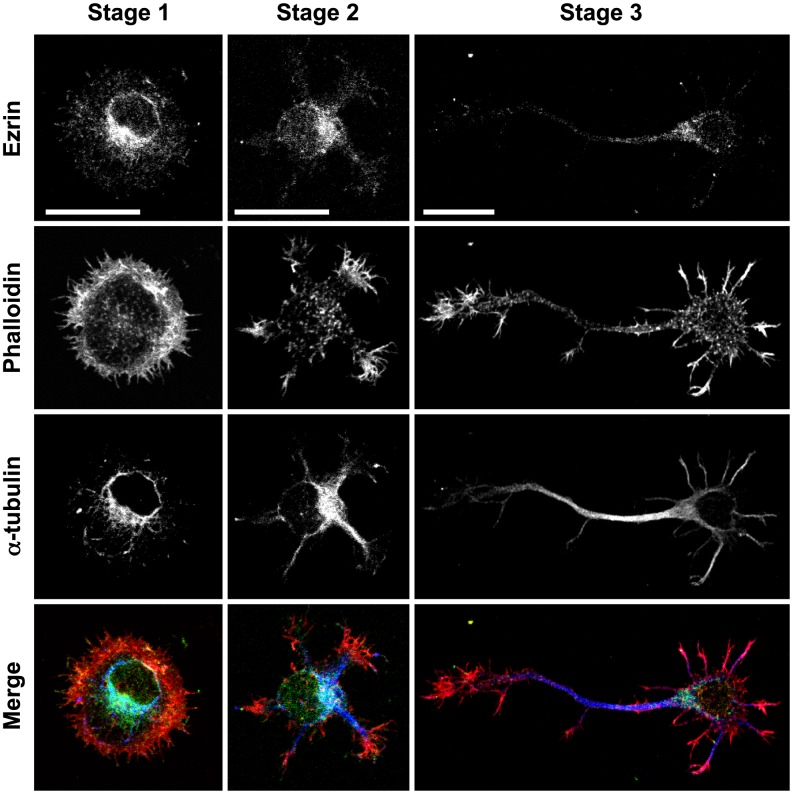
Distribution of ezrin in wild-type cultured cortical neurons at the stages 1, 2 and 3 was observed by immunofluorescence. Neurons at the stages 1, 2 and 3 were stained with an anti-ezrin antibody, rhodamine phalloidin, and an anti-α-tubulin antibody, respectively. In the bottom lane, neurons were triple stained with an anti-ezrin antibody (green), rhodamine phalloidin (red) and an anti-α-tubulin antibody (blue). Scale bars, 50 µm.

We first confirmed the distribution of ezrin in the wild-type cultured neurons by immunofluorescence (Fig. 1). Ezrin was partly overlapped with microtubules and mainly expressed in the cell body. In actin-rich region such as the lamellipodia or growth cone, only a little colocalization of ezrin with actin filaments was detected as reported in the sensory and hippocampal neurons [12], [21].

To examine the role of ezrin in the cultured cortical neurons, we performed loss-of-function studies. Previously, Antoine-Bertrand et al. [13] performed ezrin-specific siRNA treatment of cultured cortical neurons, which resulted in partial reduction of ezrin expression to 55%, and reported that the treatment impaired axon-outgrowth induced by netrin-1. Here, we newly studied the roles of ezrin in the cultured cortical neurons prepared from the *Vil2^kd/kd^* mouse embryo. A very small signal of ezrin was detected by immunofluorescence in the *Vil2^kd/kd^* neurons (Fig. S1 *A*). The same antibody recognized a single band of ezrin with a molecular mass of 80 kDa by immunoblotting in the *Vil2^+/+^* neurons. Conversely, the band was not detected in the *Vil2^kd/kd^* neurons (Fig. S1 *B*). The band was detected in the *Vil2^+/+^* neuron extracts (10 µg, 1 µg and 0.5 µg) whereas it was not detected even in 10 µg of the *Vil2^kd/kd^* neuron extracts, indicating that amount of ezrin expressed in the *Vil2^kd/kd^* neurons is less than 5% compared with the *Vil2^+/+^* neurons, which is consistent with the previous report [14]. In addition, the expression of radixin or moesin was not up-regulated in the *Vil2^kd/kd^* neurons in a compensatory manner (data not shown).

**Figure 2:**
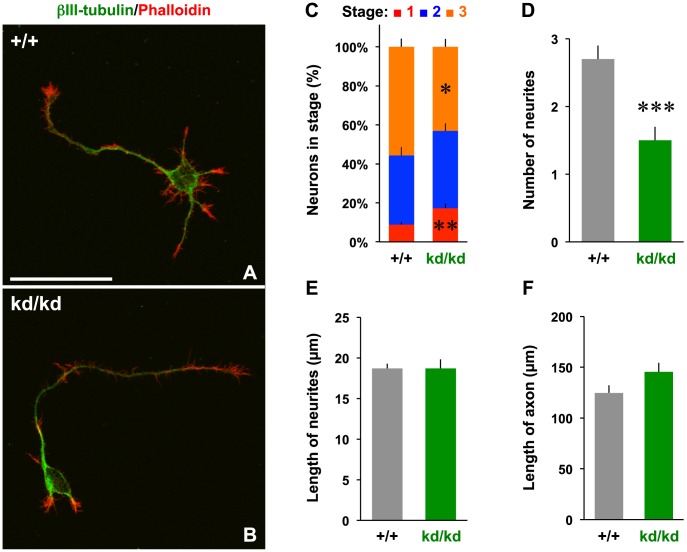
Neuritogenesis is impaired by ezrin knockdown. *A*, *B*, The *Vil2^+/+^* (*A*) and *Vil2^kd/kd^* (*B*) neurons were fixed at 2 DIV and stained with an anti-neuronal class III β-tubulin antibody (green) and rhodamine phalloidin (red). Scale bars, 50 µm. *C*, Stacked bar graph showing stage progression in the *Vil2^+/+^* (n = 153) and *Vil2^kd/kd^* (n = 162) neurons. Stage of cells were defined by the length of the longest neurite as reported previously [19]. *D-F*, Quantitation of number (*D*) and length (*E*) of neurites, and length of axon (*F*) in the *Vil2^+/+^* (gray columns, n = 50) and *Vil2^kd/kd^* (green columns, n = 50) neurons. Three independent experiments were performed. *p<0.05, **p<0.01, ***p<0.001, Student's t test. Data represent mean ± SE.

To perform the morphological analysis, we stained the cultured cortical neurons with cytoskeletal markers, anti-neuronal class III β-tubulin antibody, and rhodamine phalloidin (Fig. 2*A,B*). First, we classified and counted several stages of neurons at 48 h after plating (2 DIV). The population of stage 1 neurons was significantly increased in the *Vil2^kd/kd^* neurons compared with the *Vil2^+/+^* neurons (*Vil2^+/+^*: 8.8±1.4%, *Vil2^kd/kd^*: 17.3±2.3%, Fig. 2*C*). Conversely, the population of stage 3 neurons was significantly decreased in the *Vil2^kd/kd^* neurons (*Vil2^+/+^*: 55.8±4.2%, *Vil2^kd/kd^*: 43.1±4.1%). Moreover, the stage 3 *Vil2^kd/kd^* neurons exhibited decreases in the number of neurites (*Vil2^+/+^*: 2.7±0.2, *Vil2^kd/kd^*: 1.5±0.2, Fig. 2*D*). On the other hand, no significant differences were detected in the length of both neurites and axon (Fig. 2*E,F*). We confirmed this morphological deficit in stage 3 neurons cultured for longer period (Fig. S2 *A*,*B*). Although length of both neurites and axon was not changed (Fig. S2 *D*,*E*), the number of neurites was decreased in *Vil2^kd/kd^* neurons (*Vil2^+/+^*: 4.2±0.6, *Vil2^kd/kd^*: 1.4±0.2, Fig. S2 *C*). There were no significant differences in the number and length of neurites between the neurons prepared from radixin knockout mice and wild-type neurons (unpublished data). These results suggest that ezrin is a key player of neuritogenesis.

### Increase of RhoA activity in Vil2^kd/kd^ neurons

**Figure 3:**
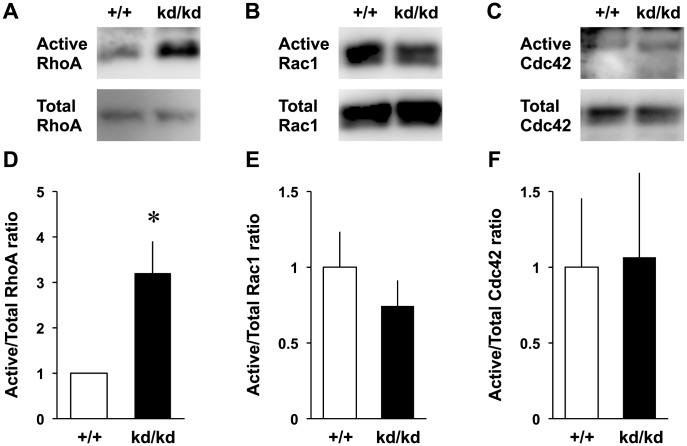
Increased RhoA activity in the Vil2^kd/kd^ neurons. *A-C*, The amounts of active and total RhoA (*A*), Rac1 (*B*) and Cdc42 (*C*) from cell lysates of the *Vil2^+/+^* and *Vil2^kd/kd^* neurons (2 DIV). Representative patterns were presented. *D-F*, The ratios of active RhoA (*D*), Rac1 (*E*) and Cdc42 (*F*) to total amount of proteins were compared between the *Vil2^+/+^* (white columns) and *Vil2^kd/kd^* (black columns) neurons. Each experiment was performed in triplicate. *p<0.05 , Student's t test. Data represent mean ± SE.

**Figure 4:**
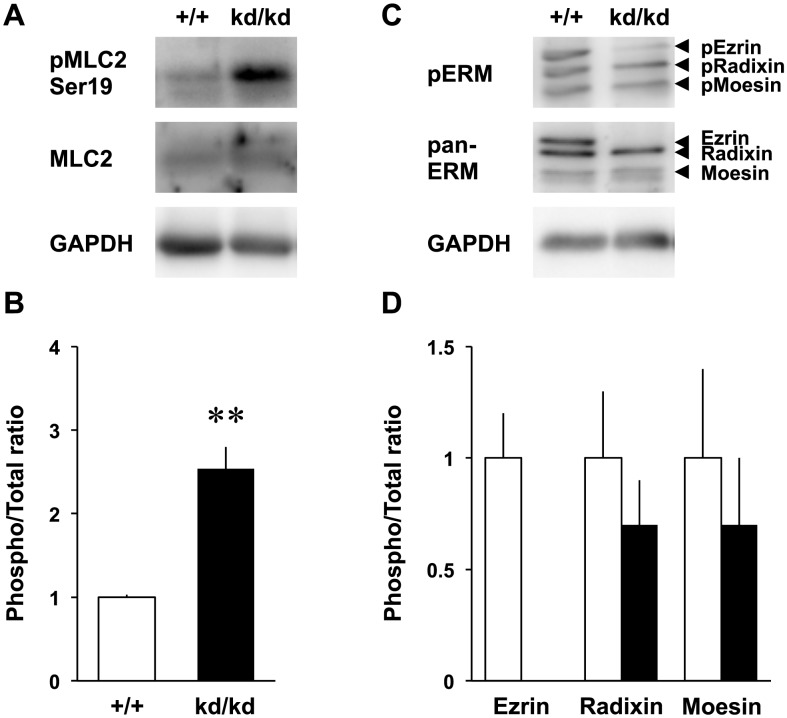
Up-regulation of MLC2 phosphorylation. *A*, Immunoblotting of the *Vil2^+/+^* and *Vil2^kd/kd^* neurons (2 DIV) using antibody recognizing phospho-MLC2 (Ser19, top), MLC2 (middle) and GAPDH (bottom), respectively. Representative blotting patterns were shown. 8 µg of cell lysate was applied onto each lane. *B*, The ratio of phosphorylated MLC2 to total MLC2 in the lysate of the *Vil2^+/+^* (white columns) and *Vil2^kd/kd^* (black columns) neurons was shown. *C*, Immunoblotting of the *Vil2^+/+^* and *Vil2^kd/kd^* neurons (2 DIV) using antibody recognizing phospho-ERM (top), pan-ERM (middle) and GAPDH (bottom), respectively. Representative blotting patterns were shown. 8 µg of cell lysate was applied onto each lane. *D*, The ratios of phosphorylated ezrin, radixin and moesin to each total protein in the lysate of the *Vil2^+/+^* (white columns) and *Vil2^kd/kd^* (black columns) neurons were shown. Each experiment was performed in triplicate. **p<0.01 , Student's t test. Data represent mean ± SE.

**Figure 5:**
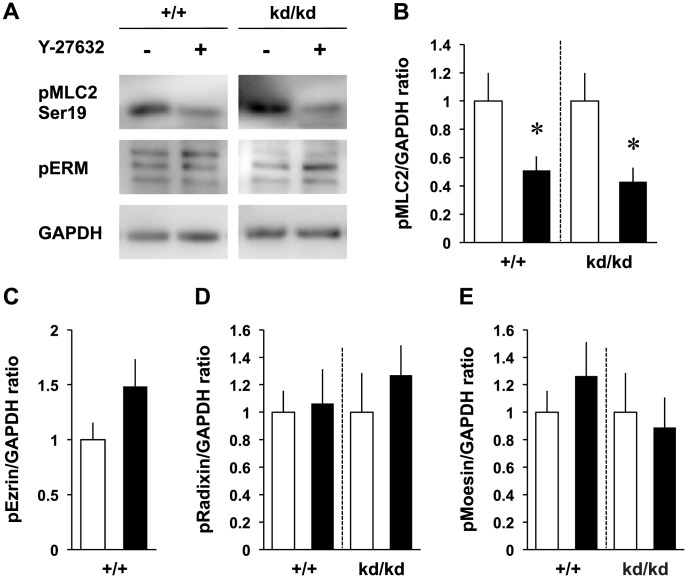
Phosphorylation was affected by Y-27632 in the MLC2, not in the ERM proteins. *A*. Immunoblotting of the DMSO- or Y-27632-treated *Vil2^+/+^* and *Vil2^kd/kd^* neurons (2 DIV) using antibody recognizing phospho-MLC2 (Ser19, top), phospho-ERM (middle) and GAPDH (bottom), respectively. Representative blotting patterns were shown. 8 µg of cell lysate was applied onto each lane. *B-E*, The ratios of phosphorylated MLC2, ezrin, radixin and moesin to GAPDH in the lysate of the DMSO-treated (white columns) and Y-27632-treated (black columns) *Vil2^+/+^* and *Vil2^kd/kd^* neurons were shown. Each experiment was performed in triplicate. *p<0.05, Student's t test. Data represent mean ± SE.

RhoA is a member of Rho family proteins that promote cell signaling pathway and cytoskeletal organization in cultured neurons [4], [22]. It was shown that RhoA activated the ERM proteins by phosphorylating their C-terminal threonine residues in 3T3 cells [23]. Conversely, ezrin was shown to be a negative regulator for RhoA because a dominant-negative form of ezrin (a mutant with its actin-binding domain being deleted) or ezrin knockout increased RhoA activity in non-neuronal cells [24], [25]. Therefore, we examined whether ezrin knockdown increases RhoA activity in the cultured cortical neurons (Fig. 3). Total amount of RhoA was not changed between the *Vil2^+/+^* and *Vil2^kd/kd^* neurons whereas GTP-bound RhoA in the *Vil2^kd/kd^* neurons was increased more than three-folds compared with the *Vil2^+/+^* neurons (Fig. 3*A,D*). In contrast to RhoA, other Rho family members, Rac1 and Cdc42 were not affected by ezrin knockdown (Fig. 3*B,E and C,F*). To confirm the involvement of RhoA activation in the neuritogenesis, we observed the phosphorylation of downstream effector in the *Vil2^kd/kd^* neurons. The phosphorylation of MLC2, which negatively regulates the actin organization, was enhanced in the *Vil2^kd/kd^* neurons (Fig. 4*A,B*). However, the immunoblotting with a polyclonal antibody that recognized all members of ERM proteins confirmed that there were no differences between the *Vil2^+/+^* and *Vil2^kd/kd^* neurons in the phosphorylation of radixin and moesin (Fig. 4*C,D*). Next, we tested whether phosphorylation of MLC2 is affected by Y-27632. Phosphorylated MLC2 was decreased in Y-27632-treated *Vil2^+/+^* neurons compared with DMSO-treated *Vil2^+/+^* neurons (Fig. 5*A,B*). Similar decrease in the phosphorylated MLC2 was also observed in Y-27632-treated *Vil2^kd/kd^* neurons, indicating that MLC2 is a downstream effector of RhoA/Rho kinase pathway. In contrast, phosphorylated ezrin, radixin and moesin were not affected by Y-27632 (Fig. 5*A,C-E*).

### Inhibition of Rho kinase and myosin II rescued neuritogenesis

**Figure 6:**
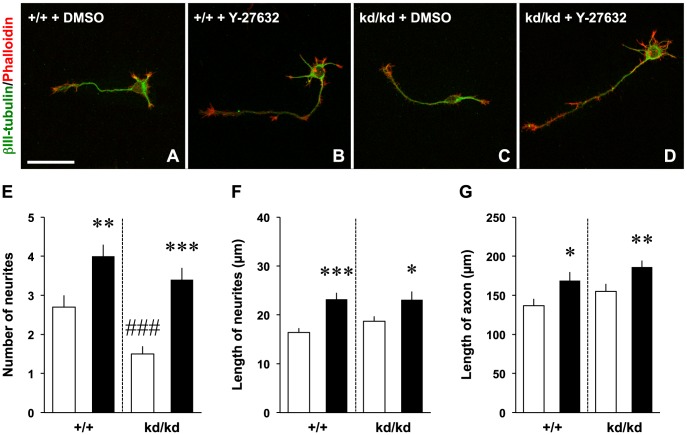
Y-27632 rescues neuritogenesis. *A-D*, The *Vil2^+/+^* and *Vil2^kd/kd^* neurons treated with DMSO (*A*,*C*) or 40 µM Y-27632 (24 h, *B*,*D*) were fixed at 2 DIV and stained with an anti-neuronal class III β-tubulin antibody (green) and rhodamine phalloidin (red). Scale bars, 50 µm. *E-G*, The number (*E*) and length (*F*) of neurites, and length of axon (*G*) were quantified in the *Vil2^+/+^* and *Vil2^kd/kd^* neurons treated with DMSO (white columns, n = 30) or 40 µM Y-27632 (black columns, n = 30). Three independent experiments were performed. *p<0.05, **p<0.01, ***p<0.001 (DMSO-treated vs. Y-27632-treated), *###p*<0.001 (DMSO-treated *Vil2^+/+^* vs. DMSO-treated *Vil2^kd/kd^*), Student's t test. Data represent mean ± SE.

**Figure 7:**
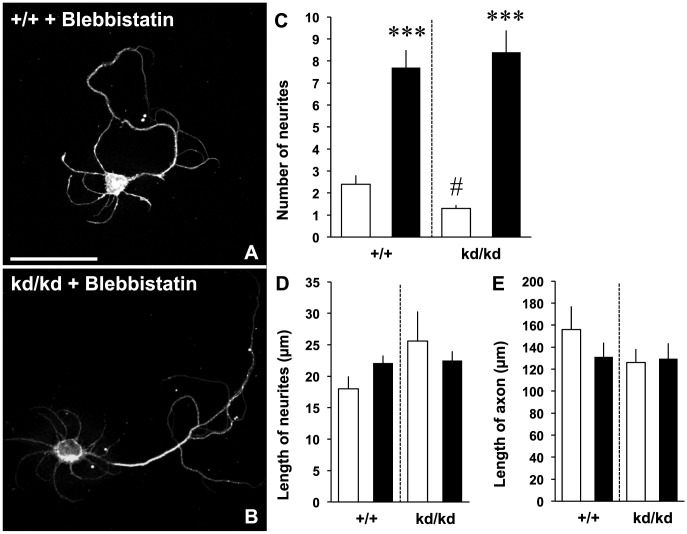
Blebbistatin rescues neuritogenesis. *A, B*, The *Vil2^+/+^* and *Vil2^kd/kd^* neurons treated with 50 µM blebbistatin (24 h) were fixed at 2 DIV and stained with an anti-neuronal class III β-tubulin antibody. Scale bar, 50 µm. *C-E*, The number (*C*) and length (*D*) of neurites, and length of axon (*E*) were quantified in the *Vil2^+/+^* and *Vil2^kd/kd^* neurons treated with DMSO (white columns, n = 10) or 50 µM blebbistatin (black columns, n = 10). Three independent experiments were performed. ***p<0.001 (DMSO-treated vs. blebbistatin-treated), *#p*<0.05 (DMSO-treated *Vil2^+/+^* vs. DMSO-treated *Vil2^kd/kd^*), Student's t test. Data represent mean ± SE.

**Figure 8:**
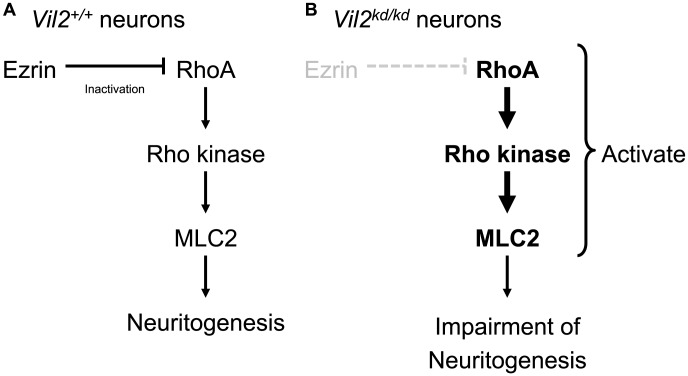
Schematic representation of the relationship between ezrin and RhoA/Rho kinase/MLC2 pathway in neuritogenesis.

We then attempted to determine whether inhibition of RhoA activation rescues neuritogenesis in the *Vil2^kd/kd^* neurons (Fig. 6*A-D*). The Rho kinase was inhibited by a specific inhibitor Y-27632, resulting in the alternation of neurite outgrowth [15], [26], [27]. Our results showed that Y-27632 was able to rescue the effect of ezrin knockdown on the neuritogenesis. In both the *Vil2^+/+^* and *Vil2^kd/kd^* neurons, the number of neurites was increased by the treatment of Y-27632 in comparison to vehicle-treated cells (Fig. 6*E*). The number of neurites was similar between Y-27632-treated *Vil2^+/+^* neurons and Y-27632-treated *Vil2^kd/kd^* neurons (Y-27632-treated *Vil2^+/+^*: 4.0±0.3, Y-27632-treated *Vil2^kd/kd^*: 3.4±.3). Similar increase in the length of neurites and axon was also observed in the *Vil2^+/+^* and *Vil2^kd/kd^* neurons (Fig. 6*F,G*). We next examined inhibition of myosin II by addition of 50 µM blebbistatin (Fig. 7*A,B*). Similar to Y-27632, treatment of blebbistatin enhanced number of neurites and rescued impairment of neuritogenesis (blebbistatin-treated *Vil2^+/+^*: 7.7±0.8, blebbistatin-treated *Vil2^kd/kd^*: 8.8±1.0, Fig. 7*C*). There were no significant differences in the length of neurites and axon between DMSO-treated neurons and blebbistatin-treated neurons (Fig. 7*D,E*). These results suggest that ezrin is essential in the neuritogenesis through down-regulation of RhoA activities (Fig. 8).

## Discussion

ERM proteins that link plasma membrane proteins and the actin cytoskeleton are expressed in various cultured neurons; hippocampal, cortical and dorsal root ganglion neurons [11], [13], [28]. Radixin and moesin, not ezrin are enriched in the actin-rich structure of growth cone such as radial striations, lamellipodial veils and filopodial extensions [29]. Radixin was abundant in the leading edge of growth cone, and the microscale chromophore-assisted laser inactivation (micro-CALI) of radixin in growth cones caused a 30% reduction of lamellipodial area in chick sympathetic neurons [30]. Moesin was phosphorylated by the treatment with glutamate in H19-7/IGF-IR cells [31]. The siRNA inhibition of moesin and inactivation of the phosphorylated ERM proteins caused reduction of active synaptic boutons induced by the glutamate treatment in the cultured hippocampal neurons [32]. In addition, in the hippocampal neurons, suppressions of both radixin and moesin, neither of ezrin-moesin nor ezrin-radixin, by the antisense oligonucleotide treatment displayed the impairment of growth cone morphology and neurite extensions [12], suggesting that radixin and moesin promote the actin organization in neuronal morphogenesis. However, among the ERM proteins, the roles of ezrin in the neuronal morphogenesis have been poorly understood.

Previously, Antoine-Bertrand et al. [13] demonstrated that the phosphorylated ERM proteins formed a complex with the receptor Deleted in Colorectal Cancer (DCC), and were associated with netrin-1-induced axon outgrowth. The netrin-1 phosphorylated the ERM proteins especially ezrin, and enhanced accumulation of the phosphorylated ERM proteins in the growth cone. The DCC-mediated neurite outgrowth in N1E-115 cells was inhibited by the expression of dominant-negative form of ezrin, in which the actin-binding domain was deleted. Moreover, ezrin siRNA treatment, which induced 55% down-regulation of its expression, impaired axon outgrowth on the netrin-1 stimulation, suggesting that ezrin is required for the axon outgrowth in the cultured cortical neurons. In our present report, we newly demonstrated that ezrin was involved in the neuritogenesis using the cultured cortical neurons prepared from the *Vil2^kd/kd^* mice embryo in which ezrin expression was down-regulated less than 5% compared with the *Vil2^+/+^* neurons. The *Vil2^kd/kd^* neurons showed decrease in the number of neurites (Fig. 2*D*) with their lengths of neurites and axon being unaltered (Fig. 2*E,F*). In the *Vil2^kd/kd^* neurons, the retardation of stage progression was also observed at the stages 1 and 3 (Fig. 2*C*). We also showed impairment of neuritogenesis in *Vil2^kd/kd^* neurons beyond 2 DIV (Fig. S2 *C*), suggesting that ezrin knockdown caused impairment, not delay, of neuritogenesis. These observations suggest that ezrin is necessary for sprouting, not extension in neurites and axon.

Previous reports suggested the possible functional redundancy of ezrin and among the ERM proteins at the cellular level [33]. However, no compensatory up-regulation of radixin and moesin was observed in the *Vil2^kd/kd^* neurons. Although we could not use ezrin knockout (*Vil2^−/−^*) mice in the present study, the expression level of ezrin in the *Vil2^kd/kd^* neurons was less than 5% compared with the *Vil2^+/+^* neurons. Therefore, it can be expected that functional role of ezrin was observed more clearly in our present loss-of-function study overcoming redundancy.

Rho family small GTPases, RhoA, Rac1 and Cdc42 coordinate actin filaments and microtubule dynamics in the neuronal morphology [4]. The inactivation of RhoA/Rho kinase pathway facilitated stability of actin filaments resulting in the initiation of neurite sprouting in the cultured hippocampal neurons and 1C11 neuronal cell line [26]. Myosin II is a downstream effector of RhoA/Rho kinase pathway and important for the neuritogenesis. It was required for the maintenance of neuronal sphere with cortical actin filaments in the early stage of neurons [7]. In fact, both length and number of minor processes were reduced by the increase of myosin II activity in the cultured cortical neurons transfected with constitutively active RhoA. In addition, the phosphorylation of MLC2 following RhoA/Rho kinase activation regulated the activity of myosin II [34], [35]. Our present findings demonstrate that the impairment of neuritogenesis was concomitant with the abnormal cytoskeletal organizations caused by activation of RhoA/Rho kinase/MLC2 pathway in the *Vil2^kd/kd^* neurons.

ERM proteins were thought to be regulators of Rho activity through interaction with Rho guanine nucleotide dissociation inhibitors or Rho GTPase-activating proteins [8], [10]. In non-neuronal cells, it was reported that down-regulation of ezrin induced activation of RhoA. Speck et al. [24] reported that activation of RhoA was observed in LLC-PK1 epithelial cells expressing a dominant-negative form of ezrin, in which the actin-binding domain was deleted. Casaletto et al. [25] demonstrated that loss of ezrin increased RhoA activity and phosphorylation of MLC2 in both colonic and small intestinal epithelia of *Vil2^−/−^* mice. In this report, we first demonstrated that ezrin down-regulated RhoA activity in neuronal cells. However, the mechanism by which ezrin inhibits RhoA activity has yet to be determined.

In Fig. 8 we propose our model in which ezrin is involved in the neuritogenesis through the regulation of RhoA/Rho kinase/MLC2 activity. In the *Vil2^+/+^* neurons, ezrin down-regulates RhoA/Rho kinase pathway, which leads to the inhibition of phosphorylation of MLC2 and activation of myosin II, and promotes the neuritogenesis (Fig. 8*A*). On the other hand, in the *Vil2^kd/kd^* neurons, activated RhoA/Rho kinase phosphorylates MLC2, which leads to the impairment of neuritogenesis (Fig. 8*B*). In fact, the inhibition of Rho kinase and myosin II rescued the morphological deficits found in the *Vil2^kd/kd^* neurons (Fig. 6*E*, 7*C*). Although treatment of blebbistatin resulted in a significant increase in number of neurites similar to treatment of Y-27632, the length of neurites and axon was not affected in both *Vil2^+/+^* and *Vil2^kd/kd^* neurons (Fig. 7*D,E*). These results suggest that myosin II activity which is controlled by phosphorylation of MLC2 and located downstream in the RhoA/Rho kinase pathway is mainly involved in neuritogenesis. These observations indicate that regulation of myosin II activity by ezrin is crucial for neuritogenesis.

In contrast to RhoA, activation of Rac1, Cdc42 and their downstream effectors such as PAK family of serine/threonine kinases promoted neuronal morphology through actin remodeling [5], [6]. It should be noted that other ERM protein, merlin, which is the neurofibromatosis type 2 gene product, inhibited neurite extensions through the inactivation of Rac1 activity in cultured cerebellar Purkinje cells [36]. Conversely, in the present study, ezrin knockdown in the cultured cortical neurons specifically activated RhoA without any effects on Rac1 and Cdc42 (Fig. 3). Future studies are necessary to understand the mechanisms how each ERM protein regulates specific Rho family proteins.

In this study, we demonstrated that ezrin facilitated neuritogenesis by regulating RhoA/Rho kinase pathway. Conversely, ERM proteins are reported to be phosphorylated by several kinases. Here, we demonstrated that there were no differences in the phosphorylation of ezrin, radixin and moesin between the DMSO-treated and Y-27632-treated neurons (Fig. 5*A,C-E*). In addition, the phosphorylation level of radixin and moesin remained unaltered in the *Vil2^kd/kd^* neurons where RhoA/Rho kinase pathway was activated (Fig. 4*C,D*). These findings suggest that endogenous phosphorylation of ERM proteins is not directly regulated by Rho kinase in the cultured cortical neurons. In fact, phosphoinositide 3-kinase (PI3 kinase), leucine-rich repeat kinase 2 (LRRK2) and protein kinase C (PKC) have been reported to be involved in phosphorylation of ERM proteins and neuronal development [32], [37], [38].

In the central nervous system, ezrin was detected in the developmental brain, rostral migratory stream and subventricular zone [39]–[41]. The expression of ezrin was also detected in radial glial cells characterized as stem cells in the intermediate zone in the prenatal human cerebrum [42]. In addition, survival and migration were affected by the inhibition of Rho kinase in neuronal stem cells in vivo [43]. Our results suggest a possible role for ezrin and its downstream effector, Rho kinase on the neuronal development in vivo.

In conclusion, our study revealed a new function of ezrin in the neuritogenesis using the *Vil2^kd/kd^* neurons. Ezrin is involved in the neuritogenesis via down-regulation of the RhoA activity and inhibition of MLC2 phosphorylation in the cultured cortical neurons.

## Supporting Information

Figure S1 **Detection of ezrin in the Vil2^+/+^ and Vil2^kd/kd^ neurons.** *A*, Immunofluorescence of the *Vil2^+/+^* and *Vil2^kd/kd^* neurons at stage 1 using an anti-ezrin antibody. Scale bar, 50 µm. *B*, Immunoblotting of cell extracts (10 µg, 1 µg, 0.5 µg or 0.2 µg) from the *Vil2^+/+^* and *Vil2^kd/kd^* neurons (2 DIV) with an anti-ezrin antibody.
(EPS)

Figure S2 **Morphological abnormalities in Vil2^kd/kd^ neurons in 4 DIV.** *A*, *B*, The *Vil2^+/+^* (*A*) and *Vil2^kd/kd^* (*B*) neurons were fixed at 4 DIV and stained with an anti-neuronal class III β-tubulin antibody. Scale bars, 50 µm. *C-E*, Quantitation of number (*C*) and length (*D*) of neurites, and length of axon (*E*) in the *Vil2^+/+^* (white columns, n = 5) and *Vil2^kd/kd^* (black columns, n = 5) neurons. Three independent experiments were performed. **p<0.01, Student's t test. Data represent mean ± SE.
(EPS)
